# The Role of Parental Mediation and Age in the Associations between Cyberbullying Victimization and Bystanding and Children’s and Adolescents’ Depression

**DOI:** 10.3390/children11070777

**Published:** 2024-06-27

**Authors:** Michelle F. Wright

**Affiliations:** Department of Psychology, Indiana State University, Terre Haute, IN 47809, USA; michelle.wright@indstate.edu; Tel.: +1-812-237-2446

**Keywords:** cyberbullying, age, gender, parental mediation, depression

## Abstract

Background/Objectives: The primary objective of this research was to assess age differences in the associations between cyberbullying victimization and bystanding and depression among 234 elementary school students (4th and 5th graders; 51% female), 363 middle school students (6th to 8th grades; 53% female), and 341 high school students (9th to 12th grades; 51% female) as well as the moderating effect of parental mediation in these relationships. Methods: Participants completed self-report questionnaires on their cyberbullying victimization and bystanding, as well as depressive symptoms. Results: The findings revealed that high levels of instructive mediation buffered against depression associated with cyberbullying victimization and bystanding across all age groups, with the strongest effects found for middle school students. Lower levels of instructive mediation and higher levels of restrictive mediation increased the positive relationships between cyberbullying victimization and bystanding and depression. Co-viewing mediation did not moderate any of the associations. Conclusions: Parental mediation of technology use has the potential to alleviate the negative consequences associated with cyberbullying victimization and bystanding. The findings highlight the importance of tailoring prevention and intervention strategies to specific age groups and to parents.

## 1. Introduction

In today’s modern society, information and communication technologies (ICTs) play a fundamental role in our daily lives, providing numerous conveniences alongside potential risks [1,2]. Cyberbullying, a notable risk associated with ICT use, has garnered attention for its detrimental effects on mental health, particularly depression [3,4,5,6]. Given the established link between cyberbullying involvement (i.e., victimization, perpetration, and witnessing) and depression, researchers are keen to identify factors that might alleviate these negative consequences. Age group and parental mediation of technology use are among the factors that could potentially influence the impact of cyberbullying involvement on mental health. However, there is a dearth of research focusing on age-related disparities in cyberbullying involvement and the potential influence of age. Moreover, although depression is commonly associated with cyberbullying across various age groups, it remains unclear whether this relationship varies based on the age group [3,4,5,6]. Therefore, the purpose of this study was to investigate age-group differences in cyberbullying victimization and witnessing. Additionally, the study sought to examine age-group differences in the moderating effect of parental mediation of technology use on the associations between cyberbullying victimization, witnessing, and depression.

### 1.1. Cyberbullying

Cyberbullying, characterized by witnessing, experiencing victimization, or perpetrating repetitive and hostile behaviors through ICTs, shares similarities with traditional face-to-face bullying [5,7,8,9]. It often involves a power imbalance between the bully and the victim, alongside a technical component. Cyberbullying behaviors encompass a range of actions (e.g., gossip, insults, and hacking) through instant messenger, social media, and text messages [10,11].

### 1.2. Depression

Cyberbullying is strongly associated with depression. Victims often experience lower levels of happiness and satisfaction and increased feelings of anger, fear, and sadness [12]. These negative consequences extend to academic and behavioral domains, with cyberbullying victims and bystanders reporting lower academic performance and an increase in internalizing problems [3,4,5,6]. However, previous research has often failed to account for face-to-face bullying involvement, a closely related factor that may confound the associations between cyberbullying involvement and depression [13,14,15].

### 1.3. Parental Mediation of Technology Use

Growing concerns about youths’ exposure to cyberbullying, either as perpetrators, victims, or bystanders, have sparked research interest in identifying the factors that may mitigate this risk. Parental mediation has emerged as a crucial factor proposed to reduce online victimization among adolescents [16,17]. Parental mediation encompasses various strategies employed by parents to manage their children’s internet and digital media usage [16]. These strategies typically fall into the following three categories: restrictive mediation, instructive mediation, and co-viewing mediation. Restrictive mediation involves controlling web and internet access, often using software installations to limit content [18]. Instructive mediation involves jointly setting rules on personal information sharing, online duration, and appropriate content consumption. Co-viewing mediation entails active participation in adolescents’ online activities, offering guidance on technology use, and recommending suitable web content.

A Spanish study on teen online safety habits found that 70.4% of parents and 67.4% of adolescents reported having internet usage rules [19]. These rules mostly involved limiting internet access days, with fewer rules addressing chatting with strangers or restricting access to violent and sexual content. Another study comprising 831 parents revealed that 60% established rules on internet usage frequency and 80% set guidelines for appropriate online behavior [20]. However, none of these rules specifically addressed cyberbullying victimization. Research has also explored how parental mediation of adolescents’ technology use influences their exposure to cyberbullying victimization. For instance, Mesch [18] found that parental mediation, particularly through monitoring and setting rules for website visitation, protected adolescents from cyberbullying. Similarly, another study found that using monitoring software and jointly establishing rules on online time reduced adolescents’ likelihood of experiencing cyberbullying victimization [12]. Overall, these findings suggest that parental mediation plays a protective role in reducing adolescents’ cyberbullying victimization [21].

### 1.4. Age

Age differences play a significant role in cyberbullying involvement, mirroring patterns observed in face-to-face bullying. Younger children may be more prone to physical forms of aggression, with verbal and relational aggression becoming more prevalent during adolescence [22]. However, the advent of ICTs has introduced new complexities, potentially increasing the susceptibility to cyberbullying among children and adolescents [3,23,24]. Although some studies suggest cyberbullying involvement peaks during early adolescence and declines thereafter, longitudinal research on age trends remains limited [25,26]. Despite some inconsistencies in predicting cyberbullying involvement based on age, factors such as technology use and types of technologies employed may better explain the variations in cyberbullying involvement than age alone [27]. Gender and age interactions also warrant further investigation in cyberbullying research.

### 1.5. The Present Study

A handful of studies have focused on the buffering effect of parental mediation of technology use in the associations between cyberbullying involvement and depression. These studies found that higher levels of parental mediation of technology use lessened the associations between cyberbullying victimization and depression [28]. Further, at lower levels of parental mediation, the positive relationship between cyberbullying victimization and depression increased. Another study revealed that high levels of instructive parental mediation protected against depression resulting from cyberbullying victimization, whereas high levels of restrictive mediation strategies worsened the relationship [29]. Thus, parental mediation of technology use can protect adolescents from the negative consequences of cyberbullying victimization and bystanding.

**Figure 1:**
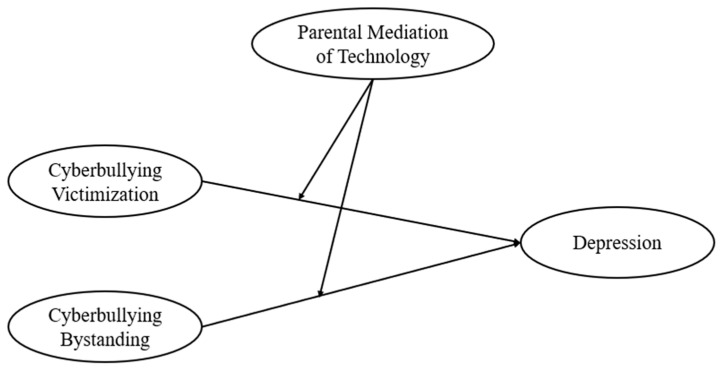
Graphical representation of the associations between cyberbullying victimization and bystanding and depression with parental mediation of technology as a moderator.

No studies have focused on how age might alter the buffering effects of parental mediation of technology use in the relationships between cyberbullying victimization and bystanding and depression. However, a body of research has found that as children age, parents implement fewer strategies and often fail to enforce those strategies [30,31,32]. In addition, older parents implement more control strategies but end up relaxing those strategies when they have conflicts with their adolescents [33]. As children become teenagers, they desire privacy concerning their technology use while also engaging in more risky behaviors. Thus, the study’s aim was to investigate the moderating effects of age in the relationships between cyberbullying involvement (i.e., victimization and witnessing) and depression (see Figure 1 for a graphical representation of the model). The research questions were as follows:

(1) How are cyberbullying victimization, bystanding, and depression related, and how do these associations vary by age, considering technology use and involvement in face-to-face bullying?
(2) Does parental mediation of technology moderate the relationships between cyberbullying victimization, bystanding, and depression and how do these moderating effects differ by age?

## 2. Materials and Methods

### 2.1. Participants

Using a random stratified sample, this study included 938 participants from middle-class suburbs of a large Midwestern city in the United States. Among these participants were 234 elementary school students, with an average age of 10.43 years (SD = 0.10). These students were distributed between 100 4th graders and 134 5th graders, with 51% of them identifying as female. Additionally, there were 363 middle school students, with an average age of 13.03 years (SD = 0.13). This group consisted of 105 6th graders, 115 7th graders, and 143 8th graders, with 53% of them reporting that they identified as female. Furthermore, the study included 341 high school students, with an average age of 16.29 years (SD = 0.67). These students were spread across 86 9th graders, 76 10th graders, 82 11th graders, and 98 12th graders, with 51% of them reporting that they were female. In terms of racial identification, 60% of the participants identified as white, 30% as Latinx, 5% as Black/African American, 1% as Asian, and 4% as other. No additional income data were collected for this study.

### 2.2. Procedures

Before commencing data collection, ethical approval was obtained in accordance with APA ethical standards. Five school districts were randomly selected from a pool of seventy-five. Three district representatives were unavailable due to prior commitments, one district did not respond, and the final district expressed interest and granted approval at the district level for the study. Recruitment involved selecting one school from a list of elementary schools, middle schools, and high schools within the school district. No incentives were offered to participants.

**Figure 2:**
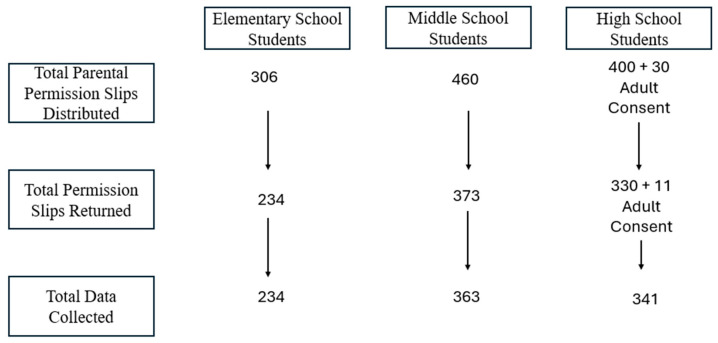
Graphical representation of the obtained final sample.

Meetings were held with school principals, followed by classroom announcements detailing the study’s purpose and expectations. Parental permission slips were sent home with students, unless they were 18 years or older, in which case informed consent documents were provided. We distributed 306 parental permission slips to elementary school students, 460 to middle school students, and 400, along with 30 informed consents, to high school students (see Figure 2 for the graphical representation of the obtained final sample).

Data collection took place during the spring of 2019. There were 10 middle schools unavailable on the day of data collection, resulting in a total of 363 middle school participants. Data were collected during regular school hours using paper questionnaires that asked participants about their age, gender, and frequency of technology use as well as questionnaires on bullying and cyberbullying involvement, depression, and parental mediation of technology. All participants agreed to participate on the day of data collection.

### 2.3. Measures

#### 2.3.1. Technology Use

A questionnaire asked participants to rate the frequency of their technology use (10 items; e.g., how often do you send text messages) on a scale of 1 (never) to 5 (all the time). Scores were combined to form a final score on technology use. Cronbach’s alphas ranged from 0.80 to 0.91.

#### 2.3.2. Face-to-Face Bullying and Cyberbullying Victimization and Bystanding

A questionnaire comprising thirty-two items was administered to assess participants’ experiences of bullying and cyberbullying victimization and bystanding (16 items each) [34]. The items covered various behaviors such as insults and unpleasant name-calling, both online and offline. Participants rated their experiences on a scale from 1 (never) to 5 (all the time), considering incidents only within the current school year. Subscale scores for victimization and bystanding were computed by averaging the relevant items. Higher scores indicated a greater involvement in bullying as victims and bystanders. Cronbach’s alphas ranged from 0.83 to 0.93 across all subscales.

#### 2.3.3. Depression

Participants’ depressive symptoms over the past two weeks were assessed using the Center for Epidemiological Studies Depression Scale, which comprises twenty items (e.g., loss of appetite and feelings of sadness) [35] rated on a scale from 0 (rarely or none of the time) to 3 (most or all of the time). Cronbach’s alphas for reliability ranged from 0.80 to 0.88.

#### 2.3.4. Parental Mediation of Technology Use

Participants completed a questionnaire assessing their perceptions of their parents’ involvement in their technology use, adapted from Arrizabalaga-Crespo et al. [36]. The questionnaire comprised three subscales. These were restrictive mediation (4 items; e.g., my parents impose a time limit on my internet usage), co-viewing mediation (3 items; e.g., my parents use the internet with me), and instructive mediation (2 items; e.g., my parents educate me about internet usage and its risks). Each of the nine items was rated on a scale from 1 (completely disagree) to 5 (completely agree). Scores for each subscale were computed by averaging the corresponding items. Cronbach’s alphas indicated good reliability (α = 0.88 for restrictive mediation, α = 0.88 for co-viewing mediation, and α = 0.85 for instructive mediation).

### 2.4. Analytical Plan

To address the study’s research questions, a single multigroup comparison structural equation model was employed utilizing the robust maximum likelihood estimator and the full information maximum likelihood approach to handle any missing data. Approximately 0.5% of the data were missing, resulting in twenty-two incomplete records, which were distributed as follows: fifteen from the elementary school, five from the middle school, and two from the high school. Additional paths were included from cyberbullying victimization and bystanding to parental mediation of technology use and to depression. Although gender was initially included as a predictor, it was found to be non-significant and was, therefore, excluded from further analyses. Two-way interactions were investigated between parental mediation of technology use and cyberbullying victimization and bystanding. The nature of these interactions was examined using simple slopes. Furthermore, technology use was accounted for in the analysis by allowing it to predict cyberbullying victimization and bystanding; face-to-face bullying victimization and bystanding were similarly controlled for by allowing the prediction of all forms of cyberbullying involvement.

## 3. Results

### 3.1. Correlations

Pearson correlations were performed for all variables in the study (Table 1). The findings revealed that instructive mediation was positively associated with co-viewing mediation among all age groups, although this variable was negatively associated with restrictive mediation, cyberbullying victimization, cyberbullying bystanding, and depression. Co-viewing mediation was not associated with restrictive mediation but it was negatively related to cyberbullying victimization, cyberbullying bystanding, and depression. Restrictive mediation was positively related to cyberbullying victimization, cyberbullying bystanding, and depression.

### 3.2. Associations between Cyberbullying Involvement, Parental Mediation, and Depression

To address the research questions, a multigroup comparison structural model analysis was performed, which demonstrated a satisfactory fit, with *χ*^2^ (1232) = 601.03, *p* = 0.12, CFI = 0.99, TLI = 0.99, RMSEA = 0.04, and SRMR = 0.03. Across all age groups, instructive mediation and co-viewing mediation were negatively associated with depression, while restrictive mediation was positively related to depression (See Table 2). In addition, cyberbullying victimization and bystanding were positively related to depression among all participants. The associations were stronger for middle school students when compared with high school and elementary school students.

Two-way interactions were examined between cyberbullying victimization and each parental mediation strategy as well as between cyberbullying bystanding and all parental mediation strategies. Probing the interaction further revealed that higher levels of instructive mediation diminished the positive relationships between cyberbullying victimization and bystanding and depression, while lower levels increased these positive relationships. On the other hand, higher levels of restrictive mediation increased the positive relationships between cyberbullying victimization and bystanding. No other significant moderating effects were found. The patterns of the associations were consistent across each age group, with stronger magnitudes among middle school students.

## 4. Discussion

This study explored the associations between cyberbullying victimization and bystanding, parental mediation strategies (including restrictive, co-viewing, and instructive approaches), and depression. Additionally, the study aimed to assess how parental mediation strategies could moderate the relationships between cyberbullying victimization, bystanding, and depression. A final aim of this research was to examine the role of age (i.e., elementary, middle, and high school age) in these associations. Given the potential protective role of parental mediation strategies, it is imperative to identify factors that may mitigate the adverse consequences of cyberbullying victimization and bystanding on children’s and adolescents’ depression. Moreover, educating parents about effective mediation strategies through intervention programs can be instrumental in addressing cyberbullying and its impacts.

Addressing the first research question, the findings revealed positive associations between cyberbullying victimization, bystanding, and depression that were consistent with prior research [37,38,39,40]. This extends the existing knowledge on the detrimental effects of cyberbullying perpetration and victimization to include cyberbullying bystanding as well. The theory of learned helplessness [41] provides a framework to understand these associations, suggesting that feelings of helplessness and lack of control when being victimized by cyberbullying and witnessing cyberbullying may contribute to negative mental health outcomes such as depression.

Instructive and co-viewing mediation strategies were negatively related to cyberbullying victimization and bystanding, while restrictive mediation showed a positive association. Restrictive mediation, characterized by setting rules without fostering an open dialogue, may hinder adolescents’ ability to develop coping mechanisms for online risks, potentially increasing their susceptibility to cyberbullying involvement. The findings regarding instructive and co-viewing mediations aligned with the existing literature on parental mediation strategies [21]. These strategies provide opportunities for parents to discuss online experiences with their children, which may help to reduce exposure to cyberbullying and its negative impacts. Instructive and co-viewing mediations also facilitate continuous communication between parents and children regarding online experiences, potentially reducing the likelihood of cyberbullying involvement.

Instructive mediation also moderated the associations between cyberbullying victimization and bystanding and depression, weakening these relationships at higher levels of instructive mediation and increasing the positive relationships at lower levels. Adolescents with parents who engage in instructive mediation may develop effective coping skills and strategies to avoid cyberbullying situations. Restrictive mediation moderated the associations between cyberbullying victimization and bystanding and depression. Higher levels of restrictive mediation strengthened these positive relationships, while lower levels did not impact them. Restrictive mediation, characterized by strict rules without open discussions, may hinder adolescents’ development of coping skills, increasing vulnerability to negative outcomes. Restrictive mediation could potentially manifest as overprotective parenting, where parents rely on fear tactics to guide their children’s online behavior, inadvertently leaving them vulnerable to negative online encounters [42,43]. Despite implementing strict rules, parents employing restrictive mediation may overlook discussions on strategies to navigate online risks [18]. Co-viewing mediation did not significantly moderate these associations, suggesting that although it may provide some social support, it may not effectively mitigate the impacts of cyberbullying.

These findings aligned with the existing literature, demonstrating that parental mediation of technology usage can mitigate adolescents’ susceptibility to cyberbullying [12,18]. Moreover, the observed buffering effect of parental mediation resonated with prior research [28]. Social support has been shown to attenuate the adverse outcomes associated with various negative online and offline encounters among adolescents [44,45]. Parental mediation of technology serves as a form of social support, wherein parents impart effective strategies to navigate and evade online risks. Additionally, ongoing discussions about online hazards and opportunities within the family dynamic foster an environment where adolescents can voice concerns and seek guidance to avoid detrimental online experiences. Although the complete avoidance of negative online encounters may not always be feasible, adolescents derive a sense of security from knowing they have their parents’ support, thereby alleviating the adjustment challenges associated with online risks, including cybervictimization.

The patterns of the associations were similar across all age groups, with the magnitudes of associations strongest for middle school students. Sevcikova and Smahel [26] discovered that early adolescents exhibited the highest rates of both perpetrating and being victimized by cyberbullying compared with younger and older age cohorts. Our study’s identification of middle school students—who fall within the early adolescent category—as having the highest levels of cyberbullying involvement aligned with Sevcikova and Smahel’s findings. However, contrasting results have been reported by other studies [46], which indicated a higher involvement in cyberbullying among different age groups. The discrepancies between these studies and ours may stem from variations in how the cyberbullying was measured, complicating direct comparisons. Our study addressed this issue by controlling for factors such as technology use and face-to-face bullying involvement, representing a methodological enhancement over prior research on age-related disparities in cyberbullying participation. Ultimately, understanding that the magnitude of the associations was strongest for middle school students highlights the importance of targeted intervention efforts for this age group that involve both adolescents and their parents.

There are some limitations to the present study that must be noted. First, the study might not be representative of children and adolescents beyond the participants included in this study. A relatively small number of schools, all from the same school district, were recruited for this study, potentially limiting how the findings could apply to other schools and school districts. Some parental permission slips were unreturned or permission was not granted for some of the potential participants and, therefore, their experiences are unknown. They might have had different experiences from the participants who had permission and who participated in the study.

Although the present study uniquely focused on investigating age disparities in the influence of parental mediation on the relationship between cyberbullying and depression, its cross-sectional nature posed challenges to our understanding of the longitudinal associations between the examined variables. Subsequent research endeavors should incorporate longer-term investigations and assess these variables at multiple time points to elucidate the temporal sequencing of parental mediation, cyberbullying involvement, and depression. Additionally, longitudinal studies tracking the same age group over time would shed light on potential changes in cyberbullying involvement, parental mediation of technology use, and depression. Moreover, such follow-up research could explore the potential roles of other variables such as poverty and attachment to parents and peers in the examined relationships. Similarly, such research should collect information regarding the socioeconomic status of the family, whether the parents live together or not, and the educational status of the parents as potential variables that could impact the associations examined in this study.

Although the present study primarily focused on depression as the outcome of cyberbullying involvement, the existing literature underscores the various negative repercussions associated with cyberbullying, including anxiety, suicidal ideation, non-suicidal self-harm, subjective health complaints, substance use, and academic difficulties. For instance, academic performance has been shown to differ between non-bully/non-victim and bully/victim groups. Future research endeavors should not only employ longitudinal designs but also examine additional outcomes linked to cyberbullying involvement to explore age disparities and the potential buffering effects of parental mediation against other adverse consequences.

## Figures and Tables

**Table 1:** Pearson correlations among all variables.

	1	2	3	4	5	6
1. Instructive Mediation	---					
2. Co-Viewing Mediation	0.20 *	---				
	0.24 *					
	0.21 *					
3. Restrictive Mediation	−0.20 *	−0.03	---			
	−0.31 ***	−0.11				
	−0.25 **	−0.02				
4. CBV	−0.26 **	−0.20 *	0.24 **	---		
	−0.39 ***	−0.26 **	0.33 ***			
	−0.30 ***	−0.22 *	0.28 ***			
5. CBW	−0.18 *	−0.18 *	0.21 **	0.26 **	---	
	−0.28 **	−0.25 **	0.30 ***	0.31 ***		
	−0.20 *	−0.18 *	0.24 **	0.28 **		
6. Depression	−0.30 ***	−0.27 **	0.29 ***	0.31 ***	0.28 **	---
	−0.35 ***	−0.30 ***	0.35 ***	0.36 ***	0.34 ***	
	−0.30 ***	−0.27 **	0.33 ***	0.30 ***	0.28 **	

CBV: cyberbullying victimization; CBW: cyberbullying witnessing. The first number corresponds with elementary school students, the second number corresponds with middle school students, and the third number corresponds with high school students. * *p* < 0.05, ** *p* < 0.01, and *** *p* < 0.001.

**Table 2:** Multigroup comparison of the associations between parental mediation, cyberbullying victimization, cyberbullying bystanding, and depression by age group.

		Depression	
School Type	Predictors	β	SE
Elementary School	Instructive Mediation (IM)	−0.25 **	0.10
	Co-Viewing Mediation (CM)	−0.18 *	0.06
	Restrictive Mediation (RM)	0.25 **	0.10
	CBV	0.27 **	0.11
	CBW	0.20 *	0.08
	IM × CBV	−0.11 *	0.04
	CM × CBV	−0.03	0.01
	RM × CBV	0.12 *	0.05
	IM × CBW	−0.10 *	0.04
	CM × CBW	−0.02	0.01
	RM × CBW	0.10 *	0.03
Middle School	Instructive Mediation (IM)	−0.30 ***	0.13
	Co-Viewing Mediation (CM)	−0.28 **	0.12
	Restrictive Mediation (RM)	0.28 **	0.12
	CBV	0.30 ***	0.13
	CBW	0.27 **	0.11
	IM × CBV	−0.20 ***	0.08
	CM × CBV	−0.01	0.01
	RM × CBV	0.21 ***	0.09
	IM × CBW	−0.18 ***	0.06
	CM × CBW	−0.02	0.01
	RM × CBW	0.19 ***	0.08
High School	Instructive Mediation (IM)	−0.25 **	0.10
	Co-Viewing Mediation (CM)	−0.20 *	0.09
	Restrictive Mediation (RM)	0.26 **	0.10
	CBV	0.28 **	0.12
	CBW	0.22 *	0.08
	IM × CBV	−0.12 *	0.03
	CM × CBV	−0.02	0.01
	RM × CBV	0.13 *	0.05
	IM × CBW	−0.11 *	0.04
	CM × CBW	−0.01	0.01
	RM × CBW	0.11 *	0.03

CBV: cyberbullying victimization; CBW: cyberbullying witnessing. The first number corresponds with elementary school students, the second number corresponds with middle school students, and the third number corresponds with high school students. * *p* < 0.05, ** *p* < 0.01, and *** *p* < 0.001.

## Data Availability

Data can be requested from the first author.
